# C-reactive protein/albumin ratio is associated with lung function among children/adolescents with cystic fibrosis: a three-year longitudinal study

**DOI:** 10.1590/1516-3180.2017.0109100917

**Published:** 2017-12-18

**Authors:** Julia Carvalho Ventura, Daniela Barbieri Hauschild, Emília Addison Machado Moreira, Letícia Cristina Radin Pereira, Anauã Franco Rosa, Eliana Barbosa, Norberto Ludwig-Neto, Julia Salvan da Rosa, Tânia Silvia Fröde, Yara Maria Franco Moreno

**Affiliations:** I MSc. Doctoral Student, Postgraduate Program on Nutrition, Universidade Federal de Santa Catarina (UFSC), Florianópolis (SC), Brazil.; II PhD. Professor, Department of Nutrition and Postgraduate Program on Nutrition, Universidade Federal de Santa Catarina (UFSC), Florianópolis (SC), Brazil.; III MSc. Doctoral Student, Department of Agricultural, Food and Nutritional Science, University of Alberta, Edmonton, Alberta, Canada.; IV BSc. Dietitian, Florianópolis (SC), Brazil.; V MSc. Dietitian, Joana de Gusmão Children’s Hospital. Florianópolis (SC), Brazil.; VI MD. Physician, Joana de Gusmão Children’s Hospital. Florianópolis (SC), Brazil.; VII MSc. Doctoral Student, Department of Clinical Analysis, Universidade Federal de Santa Catarina (UFSC), Florianópolis (SC), Brazil.; VIII PhD. Professor, Department of Clinical Analysis, Universidade Federal de Santa Catarina (UFSC), Florianópolis (SC), Brazil.

**Keywords:** Cystic fibrosis, Nutritional status, Lung diseases, C-reactive protein, Serum albumin

## Abstract

**BACKGROUND:**

Chronic lung infections, inflammation and depletion of nutritional status are considered to be prognostic indicators of morbidity in patients with cystic fibrosis. The aim of this study was to investigate the association between inflammatory markers and lung function, nutritional status and morbidity among children/adolescents with cystic fibrosis.

**DESIGN AND SETTINGS:**

Prospective three-year longitudinal study conducted in an outpatient clinic in southern Brazil.

**METHODS:**

Children/adolescents aged 1-15 years with cystic fibrosis were enrolled. Nutritional status was determined from weight-to-length and body mass index-to-age z-scores and was classified as acceptable, at risk or nutritional failure. Tumor necrosis factor-α, interleukin-1β, myeloperoxidase, C-reactive protein and C-reactive protein/albumin ratio were analyzed. Lung function was evaluated based on the forced expiratory volume in the first second and morbidity according to the number of hospitalizations for pulmonary exacerbation and infections by *Pseudomonas aeruginosa*. Lung function, nutritional status and morbidity were the outcomes. Odds ratios and 95% confidence intervals were to evaluate the effect of baseline inflammatory markers on the clinical outcomes after three years of follow-up and p-values < 0.05 were considered significant.

**RESULTS:**

We evaluated 38 children/adolescents with cystic fibrosis: 55% female; median age (with interquartile range), 3.75 years (2.71-7.00). Children/adolescents with high C-reactive protein/albumin ratio at baseline had odds of 18 (P = 0.018) of presenting forced expiratory volume in the first second ≤ 70% after three years. The other inflammatory markers were not associated with the outcomes.

**CONCLUSION:**

C-reactive protein/albumin ratio was associated with forced expiratory volume in the first second ≤ 70% after three years.

## INTRODUCTION

Cystic fibrosis is an autosomal recessive genetic disease resulting from abnormal functioning of the transmembrane protein that regulates ionic transport: the cystic fibrosis transmembrane conductance regulator. It leads to a set of manifestations and complications, such as the presence of thick, viscous secretions and obstruction of exocrine gland ducts, thus compromising the respiratory and digestive systems.^1^

Lung disease is the fundamental cause of morbidity and mortality in cystic fibrosis. Individuals affected by cystic fibrosis rarely demonstrate respiratory symptoms at birth. However, within a short time, the combination of obstruction, inflammation and infection begins to exert a negative effect on lung growth, structure and function.^1^^,^^2^

Bacterial infection, due to *Pseudomonas aeruginosa*, *Staphylococcus aureus* or the *Burkholderia cepacia* complex, for example, can adapt to the lung environment. It may affect the host’s defenses through bacterial adherence to the thick mucus or through secretion of extracellular polysaccharides, thereby favoring chronic bacterial infection and providing continuous stimulation of an inflammatory response.^3^

Persistent neutrophil infiltration leads to hyperactivation of nuclear transcription factor-kappa B, which promotes overexpression of pro-inflammatory cytokines such as tumor necrosis factor-α and interleukin-1β.^4^ The continuous inflammatory response and persistent inflammation in individuals with cystic fibrosis lead to an imbalance in cytokine levels, thus stimulating hepatic production of acute-phase proteins, such as C-reactive protein.^5^ High levels of C-reactive protein are associated with lower forced expiratory volume in the first second in individuals with cystic fibrosis.^6^ In addition to C-reactive protein levels, the C-reactive protein/albumin ratio can be evaluated and used as an inflammatory-nutritional factor.^7^

The systemic inflammatory response is associated with a continuous catabolic response, which exerts an effect on fat-free mass and leads to reduced skeletal muscle mass and wastage of inspiratory muscles.^8^ Thus, nutritional status, body composition and inflammatory conditions are considered to be prognostic indicators of morbidity and mortality among individuals with cystic fibrosis.^9^

## OBJECTIVE

The aim of the present study was to analyze the association between inflammatory markers (tumor necrosis factor-α, interleukin-1β, myeloperoxidase, C-reactive protein and C-reactive protein/albumin ratio) and lung function, nutritional status and morbidity among children/adolescents with cystic fibrosis.

## METHODS

### Study design, participants and ethics

A three-year prospective longitudinal study was conducted between April 2009 and December 2012 involving children/adolescents aged 1-15 years with a diagnosis of cystic fibrosis through the sweat test or newborn screening. The participants were recruited at an interdisciplinary cystic fibrosis outpatient clinic in southern Brazil.

A non-probabilistic convenience sample based on temporal saturation that was recruited between April 2009 and July 2010 was used. The inclusion criteria were that the subjects needed to be 1-15 years of age and have a diagnosis of cystic fibrosis that had been confirmed through an abnormal sweat test (chloride in sweat ≥ 60 mmol/l).^10^ The exclusion criteria were the presence of pulmonary exacerbation, fever, trauma, inflammatory disease (asthma, intestinal inflammatory disease and rheumatic disease), psychiatric disorder, degenerative condition, cardiovascular disease, diabetes, glucose intolerance, kidney failure, primary or secondary immunodeficiency, and use of anti-inflammatory and/or immunosuppressive drug treatment. Children/adolescents with a negative sweat test or who discontinued their treatment at the hospital were also excluded.

Considering the lack of a cutoff point for the inflammatory markers, a group without cystic fibrosis was selected among patients at the child care clinic in order to determine the cutoff points for tumor necrosis factor-α, interleukin-1β, myeloperoxidase, C-reactive protein and C-reactive protein/albumin ratio. The inclusion criteria for the group without cystic fibrosis comprised absence of a diagnosis of cystic fibrosis and being within the range of good health and ideal nutrition, as determined by a weight-to-length z-score or body mass index-to-age z-score between -2 and +2.^11^

Data collection, at the baseline of the study, involved determination of anthropometric data, lung function and inflammatory markers. Clinical data (use of enzymes) and patient identification were also collected at the baseline of the study, from patient charts. Outcome data on lung function, nutritional status and morbidity (positive cultures, number of hospitalizations and respective causes) were collected from patient charts after a three-year follow-up.

The study was approved by the local institutional review board (human research ethics committee). Informed consent was obtained from the parents or guardians of all the patients enrolled in the study.

### Nutritional status evaluation

Weight was determined using a digital pediatric scale (Filizola, São Paulo, Brazil) for children up to two years of age and a standard scale (Balmak model BK 50 F, São Paulo, Brazil) for individuals over two years of age. Length was measured using a children’s stadiometer with a movable plate (Sanny, São Paulo, Brazil) for children up to two years of age and height was determined using a length board (Alturaexata, Minas Gerais, Brazil) for individuals over two years of age.^12^

Weight-to-length percentiles were calculated for children up to two years of age and body mass index-to-age percentiles for individuals over two years of age, using reference curves.^12^ Nutritional status was classified based on the recommendations of the American Cystic Fibrosis Foundation,^13^ as nutritional failure (< 10^th^ percentile), at risk (10^th^ to 25^th^ percentile) or acceptable (> 25^th^ percentile). Nutritional status was considered ideal when weight-to-length was ≥ 50^th^ percentile for children up to two years of age, and when body mass index-to-age was ≥ 50^th^ percentile for individuals over two years of age.^14^

### Lung function evaluation

A spirometer (Renaissance Spirometry System, Puritan-Bennet Corporation, North Carolina, USA) was used for individuals aged six years and over, since children younger than six years have lower capacity to perform voluntary respiration maneuvers in an efficient manner.^15^ Spirometry was supervised by a trained professional, following the recommendations of the American Thoracic Society (Puritan-Bennett Corporation, North Carolina, USA). Respiratory obstruction was evaluated based on the forced expiratory volume in the first second, taking the forced expiratory volume in the first second to be ≤ 70%.^16^

### Bacterial evaluation

After a state of oral hygiene had been achieved, samples of oropharyngeal secretion were collected using a sterile swab that was introduced into the oropharyngeal cavity following deep coughing effort. These samples were processed using the method described by Gilligan et al.^17^ All sample collection was performed by a trained professional. The evaluation was performed under a microscope (Nikon E200, Tokyo, Japan), using the Gram method.^18^

### Inflammatory markers

Peripheral venous blood (10 ml) was collected from the cubital vein after the patient had been fasting for 10 hours. Serum was obtained, separated into aliquots and stored at -80 °C until processing to determine the study parameters.

The inflammatory markers analyzed were tumor necrosis factor-α, interleukin-1β, myeloperoxidase and C-reactive protein levels. The serum levels of tumor necrosis factor-α and interleukin-1β were determined using an enzyme-linked immunosorbent assay.^19^ The results were expressed as pg/ml. The C-reactive protein level was determined using the nephelometric method (Behring Nephelometer BN 2*,* Berlin, Germany) and the data were expressed as mg/dl. Myeloperoxidase activity was measured using the colorimetric method developed by Rao et al.^20^ and was read with absorbance at 450 nm in an enzyme-linked immunosorbent assay reader (Organon-Tecknica, New Jersey, USA); the results were expressed as mU/ml. The albumin level was determined using bromocresol green with the aid of a specific kit (Labtest Diagnóstica, Labtest, Minas Gerais, Brazil), using a colorimetry assay, and the data were expressed as g/dl.^21^ The C-reactive protein/albumin ratio was determined by dividing the C-reactive protein values by the albumin values, and the data were expressed as mg/dl:g/dl.^22^

### Morbidity, clinical condition and pancreatic function

Morbidity was evaluated based on the frequency of hospitalizations due to pulmonary exacerbation^23^ and infections by *Pseudomonas aeruginosa* over the three-year period. Presence of infection was established as a count greater than 10^4^ colony-forming units.

The Shwachman-Kulczycki score was used to classify clinical condition, with points attributed to general activities, physical examination, nutritional status and radiological findings. A score of ≥ 86 points was considered to be excellent, 71 to 85 good, 56 to 70 average, 41 to 55 poor and ≤ 40 severe.^24^ In accordance with the standard medical regimen, patients who used pancreatic enzymes were classified as having pancreatic insufficiency.^25^

### Statistical analysis

The statistical analysis was performed using the STATA software, version 11.0 (Stata Corp., Texas, USA). Quantitative variables were expressed as means and standard deviations or as medians and interquartile ranges, depending on the symmetry. Nominal variables were expressed as percentages and 95% confidence intervals. The t test or Mann-Whitney test, when appropriate, was used to determine differences between means. Pearson’s or Fisher’s chi-square test, when appropriate, was used to determine the strength of associations between the categorical variables. Odds ratios and 95% confidence intervals were calculated to evaluate the effect of baseline inflammatory markers on the clinical outcomes after three years of follow-up. Inflammatory markers were classified as “adequate” or “high”, using the 90^th^ percentile in the group without cystic fibrosis as the cutoff point. P-values < 0.05 were considered significant.

## RESULTS

**Figure 1:**
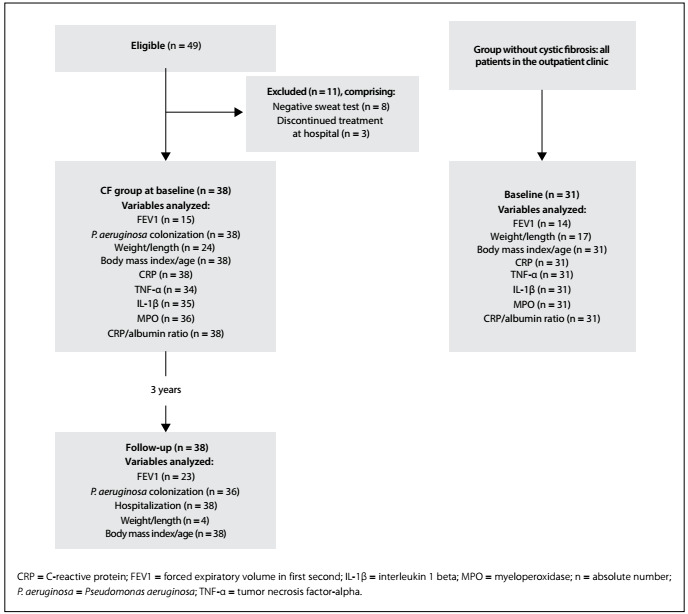
Flowchart of selection of children/adolescents with (CF group) and without cystic fibrosis in follow-up, 2009-2012.

At the baseline of the study, 49 children/adolescents with cystic fibrosis were recruited, and 38 of them met the eligibility criteria (Figure 1).

The median age of the children with cystic fibrosis (n = 38) was 3.75 years (interquartile range: 2.71 to 7.00 years) and 55% were female. The median age at which cystic fibrosis was diagnosed was 3.50 months (interquartile range: 2.00 to 11.00 months). Their mean forced expiratory volume in the first second was 76.67% ± 19.90% and the Shwachman-Kulczycki score was 86.76 ± 17.92 (Table 1).

**Table 1:** Clinical and demographic characterization at baseline of study, among children/adolescents undergoing clinical follow-up at interdisciplinary cystic fibrosis outpatient clinic

Variable	Cystic fibrosis group (n = 38)		Group without cystic fibrosis (n = 31)		P-value
		95% CI		95% CI	
Sex^1^					
Male	17 (44.74)	(28.17; 61.30)	12 (38.71)	(20.55; 56.87)	0.614^4^
Female	21 (55.26)	(38.70; 71.82)	19 (61.29)	(43.13; 79.45)	
Age (years)^2^	3.75 (2.71; 7.00)	-	4.62 (3.04; 8.91)	-	0.242^5^
Age at diagnosis (months)^2^	3.50 (2.00; 11.00)	-	-	-	
Mutation^1^					
Delta F508 homozygote	7 (18.42)	(5.51; 31.33)	-	-	-
Delta F508 heterozygote	17 (44.74)	(28.17; 61.30)	-	-	
Other mutation	6 (15.79)	(3.64; 27.93)	-	-	
Not evaluated	8 (21.05)	(7.47; 34.63)	-	-	
Culture from oropharyngeal secretion^1^					
Negative	16 (42.11)	(25.66; 58.55)	12 (100)	-	-
*P. aeruginosa*	12 (31.58)	(11.65; 40.98)	-	-	
Other*	10 (26.32)	(16.09; 47.06)	-	-	
Nutritional status^1^					
Acceptable (> 25^th^ percentile)	22 (57.89)	(41.44; 74.34)	-	-	-
At risk	11 (28.95)	(13.84; 44.05)	-	-	
Nutritional failure	5 (13.16)	(1.89; 24.41)	-	-	
Nutritional status (W/L or BMI/A)^1†^					
< 50^th^ percentile	25 (65.79)	(49.99; 81.59)	12 (38.71)	(22.70; 57.59)	0.025^4^
≥ 50^th^ percentile	13 (34.21)	(18.41; 50.01)	19 (61.29)	(42.41; 77.29)	
FEV1 (%)^3^	76.67 (± 19.90)	(65.64; 87.69)	88.57 (± 10.0)	(82.80; 94.34)	0.054^6^
Pancreatic failure^1^	29 (76.32)	(62.15; 90.47)	-	-	-
Shwachman-Kulczycki score^3^	86.76 (± 17.92)	(80.78; 92.73)	-	-	-

*P. aeruginosa = Pseudomonas aeruginosa*; BMI/A = body mass index-to-age; CI = confidence interval; FEV1 = forced expiratory volume in first second; W/L = weight-to-length; *others = *Staphylococcus aureus* and *Burkholderia cepacia;* ^†^weight-to-length < 2 years of age; body mass index-to-age ≥ 2 years of age; ^1^n (%); ^2^median (interquartile range); ^3^mean (± standard deviation); ^4^chi-square; ^5^Mann-Whitney; ^6^Student t test.

In the group without cystic fibrosis (n = 31), the median age was 4.62 years (interquartile range: 3.04 to 8.91) and 61% were female. There were no significant differences in sex and age between the groups at the baseline (Table 1).

Regarding the classification of nutritional status based on the criteria of the American Cystic Fibrosis Foundation (2009), 22 children/adolescents (58%) were classified as having acceptable nutritional status (> 25^th^ percentile) and 25 (66%) were considered to have ideal nutritional status (≥ 50^th^ percentile) according to their weight-to-length or body mass index-to-age percentile (Table 1). In comparing the nutritional status between groups, 19 (61%) of the children/adolescents without cystic fibrosis were considered to have ideal nutritional status (≥ 50^th^ percentile) while 13 (34%) of the children/adolescents with cystic fibrosis were considered to have ideal nutritional status (≥ 50^th^ percentile), according to the weight-to-length or body mass index-to-age percentile (P = 0.025) (Table 1).

At the baseline of the study, higher median interleukin-1β and myeloperoxidase values were found in the cystic fibrosis group than in the group without cystic fibrosis (P < 0.001). The median C-reactive protein/albumin ratio was also higher in the cystic fibrosis group, but the difference was not significant (P = 0.077) (Table 2).

**Table 2:** Characterization of inflammatory markers at baseline of study among children/adolescents undergoing clinical follow-up at interdisciplinary cystic fibrosis outpatient clinic

Variable	Cystic fibrosis group (n = 38)	Group without cystic fibrosis (n = 31)	P-value
	Median (IQR)	Median (IQR)	
TNF-α (pg/ml)	36.70 (1.63; 63.67)	48.88 (40.18; 58.16)	0.085
IL-1β (pg/ml)	12.55 (8.36; 39.98)	4.43 (2.38; 4.73)	< 0.001
MPO (mU/ml)	395.34 (285.42; 488.07)	198.47 (177.95; 199.96)	< 0.001
CRP (mg/dl)	4.45 (1.60; 10.09)	2.80 (2.30; 4.60)	0.110
CRP/albumin (mg/dl:g/dl)	1.36 (0.36; 2.77)	0.70 (0.40; 1.10)	0.077

CRP = C-reactive protein; IL-1β = interleukin-1 beta; IQR = interquartile range; MPO = myeloperoxidase; n = absolute number; TNF-α = tumor necrosis factor-alpha. Mann-Whitney test.

High C-reactive protein/albumin ratio at the beginning of the study was associated with forced expiratory volume in the first second ≤ 70% after three years of follow-up, in comparison with those with a normal C-reactive protein/albumin ratio, with odds of 18.00 (95% confidence interval: 1.63; 198.51) (P = 0.018). The C-reactive protein/albumin ratio was not associated with hospitalization (odds ratio: 2.00; 95% confidence interval: 0.52; 7.70), or with a positive culture for *Pseudomonas aeruginosa* (odds ratio: 0.65; 95% confidence interval: 0.10; 4.14), or with body mass index-to-age < 50^th^ percentile (odds ratio: 1.96; 95% confidence interval: 0.42; 9.10). Tumor necrosis factor-α, interleukin-1β, myeloperoxidase and C-reactive protein levels were not associated with lung function, hospitalization, positive culture for *Pseudomonas aeruginosa* or nutritional status. However, patients with high myeloperoxidase activity and adequate tumor necrosis factor-α levels at the baseline had positive cultures for *Pseudomonas aeruginosa* after three years (Table 3).

**Table 3:** Bivariate association between inflammatory markers at baseline of study and clinical outcomes after three years of follow-up among children/adolescents at an interdisciplinary cystic fibrosis outpatient clinic

Variables	FEV1 ≤ 70 %		Hospitalization		Positive culture for *Pseudomonas aeruginosa*		BMI/A < 50^**th**^ percentile	
	OR (95% CI)	P-value	OR (95% CI)	P-value	OR (95% CI)	P-value	OR (95% CI)	P-value
TNF-α (pg/ml)								
Adequate	1.00	0.952	1.00	0.437	1.00	NA	1.00	0.459
High	1.08 (0.08; 14.41)		1.89 (0.38; 9.39)		-		0.53 (0.10; 2.88)	
IL-1β (pg/ml)								
Adequate	1.00	0.297	1.00	0.689	1.00	0.094	1.00	0.942
High	0.37 (0.06; 2.37)		0.75 (0.18; 3.13)		0.20 (0.03; 1.31)		1.06 (0.21; 5.37)	
MPO (mU/ml)								
Adequate	1.00	0.571	1.00	0.631	1.00	NA	1.00	0.329
High	0.43 (0.02; 8.04)		0.60 (0.07; 4.83)		-		2.87 (0.34; 23.92)	
CRP (mg/dl)								
Adequate	1.00	0.250	1.00	0.912	1.00	0.760	1.00	0.393
High	2.93 (0.47; 18.33)		0.93 (0.23; 3.64)		0.75 (0.12; 4.76)		1.96 (0.42; 9.10)	
CRP/albumin (mg/dl:g/dl)								
Adequate	1.00	0.018	1.00	0.314	1.00	0.652	1.00	0.393
High	18.00 (1.63; 198.51)		2.00 (0.52; 7.70)		0.65 (0.10; 4.14)		1.96 (0.42; 9.10)	

BMI/A = body mass index-to-age; CI = confidence interval; CRP = C-reactive protein; FEV1 = forced expiratory volume in first second; IL-1β = interleukin-1 beta; MPO = myeloperoxidase; NA = not applicable; OR = odds ratio; TNF-α = tumor necrosis factor-alpha. Fisher’s exact test.

## DISCUSSION

Associations between the C-reactive protein/albumin ratio and lung function, nutritional status, positive culture for *Pseudomonas aeruginosa* and hospitalization among patients with cystic fibrosis have been little investigated. In the present study, children/adolescents with higher C-reactive protein/albumin ratio at the baseline had higher odds of forced expiratory volume in the first second ≤ 70% after three years of follow-up.

Unlike monitoring using sputum, the advantage of systemic monitoring of inflammation via blood sampling is that it has the potential to reflect the inflammatory status throughout the lungs, as opposed to in just one region.^26^ In the present study, we evaluated serum biomarkers in children/adolescents with cystic fibrosis and found that the interleukin-1β and myeloperoxidase levels were significantly higher in the cystic fibrosis group than in the group without cystic fibrosis. However, there was no difference in tumor necrosis factor-α levels. On the other hand, a previous study reported that the tumor necrosis factor-α level was significantly higher in the cystic fibrosis group than in a control group, which might have resulted from bacterial infections, such as with *Staphylococcus aureus*, or from other microorganisms.^27^

Situations of increased inflammation in cystic fibrosis patients are well known, and the clinical course of the lung disease in cystic fibrosis cases probably depends on the nature and degree of the inflammatory response.^27^ In a study on 35 children with cystic fibrosis, aged 6 to 15 years, the levels of tumor necrosis factor-α, interleukin-1β and IL-6 in the sputum changed over a three-year period and a single determination of these markers had predictive value for a subsequent percentage decline in predicted forced expiratory volume in the first second.^28^ However, in the present investigation, C-reactive protein, tumor necrosis factor-α, interleukin-1β levels and myeloperoxidase activity at the baseline were not associated with forced expiratory volume in the first second ≤ 70% or hospitalizations due to pulmonary exacerbation after three years of follow-up.

No significant associations were found between inflammatory markers and colonization by *Pseudomonas aeruginosa* after three years. However, high cytokine concentrations are usually found in infected cystic fibrosis patients.^27^ In a previous study, conducted on 20 adults with cystic fibrosis, those with infection by *Pseudomonas aeruginosa* in association with decreased lung function and presence of systemic inflammatory response, with increased levels of tumor necrosis factor-α and C-reactive protein, experienced chronic episodes of pulmonary exacerbation.^29^ Chronic inflammatory diseases can also lead to persistent increases in the serum levels of C-reactive protein.^30^ In a cross-sectional study on 58 adults with cystic fibrosis in which the relationship between inflammatory markers in the plasma and both morbidity and the number of hospitalizations was investigated, significant associations with high plasma or serum levels of IL-6, interleukin-1β and C-reactive protein were found.^31^

*Pseudomonas aeruginosa* is one of the most prevalent microorganisms isolated in patients with cystic fibrosis. Once established, *Pseudomonas aeruginosa* infections are difficult to eradicate and have been associated with declines in lung function. Therefore, aggressive antibiotic therapy is recommended as an eradication protocol, and this is usually successful.^32^ Although C-reactive protein levels usually decrease from the beginning to the end of the eradication protocol,^33^ the present study (in which inflammatory markers were evaluated at the baseline) showed that the C-reactive protein/albumin ratio was associated with forced expiratory volume in the first second ≤ 70% after three years of follow-up. This indicates that higher levels of C-reactive protein and/or lower levels of albumin were present even in children/adolescents with *Pseudomonas aeruginosa* colonization who were being treated using eradication protocols.

C-reactive protein has biological relevance since its serum levels increase within six hours in response to infection and/or inflammation, and its circulating levels decrease rapidly when the stimulus is removed.^26^ Albumin levels are associated with the chronic nature of diseases, and represent the inflammatory status. However, evaluation of this marker alone can create bias because albumin levels are affected by poor nutritional and chronic inflammatory status.^7^ Therefore, higher levels of C-reactive protein generally suggest an acute infectious or inflammatory process, whereas low albumin is more frequently associated with chronic diseases and is often associated with nutritional deficiency.^34^ Use of these markers is feasible because they are readily measured in serum. Facilities for automated assays are available in most clinical laboratories and these markers are often determined during hospital-based follow-ups.^26^ Thus, the combination of C-reactive protein and albumin allows evaluation of an inflammatory-nutritional factor, and the C-reactive protein/albumin ratio may be an indicator of a stronger inflammatory response.^7^ In the present investigation, a significant association was found between the C-reactive protein/albumin ratio and forced expiratory volume in the first second ≤ 70% after three years of follow-up. There is a lack of studies evaluating the C-reactive protein/albumin ratio among children/adolescents with cystic fibrosis. In a study on 334 adults with severe sepsis or septic shock, it was concluded that the C-reactive protein/albumin ratio at the time of discharge from the intensive care unit was correlated with the long-term prognosis (up to 90 days) after an episode of sepsis.^35^

The present study had certain limitations. The sample size was defined using a non-probabilistic convenience method, and this has limitations, since the results cannot be extrapolated to the population. The study had a small sample size and the evaluation on lung function decreased the sample size because this evaluation can only be performed on children aged six years and over.

Moreover, there was no preexisting cutoff point for the inflammatory markers and the systemic markers used may not have reflected pulmonary changes, thereby leading to underestimation of the results. However, the subjects were recruited at a reference center for treatment of cystic fibrosis in the state of Santa Catarina, Brazil, and therefore this sample represents the children/adolescents with cystic fibrosis in the state of Santa Catarina. A cutoff point for the inflammatory markers was established based on a healthy group without cystic fibrosis and, although this group without cystic fibrosis was not matched for sex and age with the cystic fibrosis group, we believe that the serum concentrations of the inflammatory markers were not influenced by the lack of matching between the groups.

No previous studies in which the C-reactive protein/albumin ratio was evaluated among children/adolescents with cystic fibrosis were found, although determination of C-reactive protein and albumin levels forms part of routine hospital evaluations. Multicenter studies need to be conducted in order to evaluate the applicability of the C-reactive protein/albumin ratio among children/adolescents with cystic fibrosis in clinical practice.

## CONCLUSION

In the present study, there was no association between inflammatory markers with nutritional status and morbidity. A high C-reactive protein/albumin ratio was associated with forced expiratory volume in the first second ≤ 70% after three years of follow-up. Studies with larger sample sizes are necessary in order to better explore this association.
